# Can physics-informed neural networks beat the finite element method?

**DOI:** 10.1093/imamat/hxae011

**Published:** 2024-05-23

**Authors:** Tamara G Grossmann, Urszula Julia Komorowska, Jonas Latz, Carola-Bibiane Schönlieb

**Affiliations:** Department of Applied Mathematics and Theoretical Physics, University of Cambridge, Wilberforce Road, Cambridge CB3 0WA, UK; Department of Computer Science and Technology, University of Cambridge, 15 JJ Thomson Avenue, Cambridge CB3 0FD, UK; Department of Mathematics, University of Manchester, Alan Turing Building, Oxford Road, Manchester M13 9PL, UK; Department of Applied Mathematics and Theoretical Physics, University of Cambridge, Wilberforce Road, Cambridge CB3 0WA, UK

**Keywords:** partial differential equations, finite element method, deep learning, physics-informed neural networks

## Abstract

Partial differential equations (PDEs) play a fundamental role in the mathematical modelling of many processes and systems in physical, biological and other sciences. To simulate such processes and systems, the solutions of PDEs often need to be approximated numerically. The finite element method, for instance, is a usual standard methodology to do so. The recent success of deep neural networks at various approximation tasks has motivated their use in the numerical solution of PDEs. These so-called physics-informed neural networks and their variants have shown to be able to successfully approximate a large range of PDEs. So far, physics-informed neural networks and the finite element method have mainly been studied in isolation of each other. In this work, we compare the methodologies in a systematic computational study. Indeed, we employ both methods to numerically solve various linear and nonlinear PDEs: Poisson in 1D, 2D and 3D, Allen–Cahn in 1D, semilinear Schrödinger in 1D and 2D. We then compare computational costs and approximation accuracies. In terms of solution time and accuracy, physics-informed neural networks have not been able to outperform the finite element method in our study. In some experiments, they were faster at evaluating the solved PDE.

## 1. Introduction

Partial differential equations (PDEs) are a corner stone of mathematical modelling and possibly applied mathematics itself. They are used to model a multitude of physical (Lin & Segel, 1988), biological (Clifford Henry Taubes, 2008), socioeconomic (Burger *et al.*, 2014) and financial (Heston, 2015) systems and processes. Beyond classical modelling, PDEs can describe the evolution of certain stochastic processes (Risken, 1996), be used in image reconstruction (Rudin *et al.*, 1992), as well as for filtering (Kushner, 1964) and optimal control (Bellmann, 1954) of dynamical systems.

The underlying idea is always the same: we aim to represent some process through a function that describes the behaviour of the process in space and time. The PDE is then a collection of laws that this function is supposed to satisfy. An obvious questions is whether these laws along with suitable initial and boundary conditions are actually sufficient to uniquely specify this function (Lawrence, 2010). Once uniqueness or even well-posedness (Hadamard, 1902) has been established, the question is how to find the function satisfying these laws that will ultimately be the mathematical model. When referring to PDEs in the following, we assume that they have suitable initial and boundary conditions.

In many practical situations, it is impossible to find closed-form solutions and they need to be found numerically. Throughout the last decades, several numerical methods have been proposed and analysed to solve PDEs, especially the *finite element method* (FEM) (Courant, 1943; Hrennikoff, 2021) that may be the standard methodology for a huge class of PDEs. Other techniques are, e.g. the finite difference (Iserles, 2008), finite volume (Eymard *et al.*, 1997) and the spectral element (Patera, 1984) method. These approaches are usually well understood from a theoretical perspective: there are existing error estimators, as well as convergence and stability guarantees. Moreover, the given discretized problems are often in a form that can easily be solved numerically: relying on large, but sparse linear systems or on Newton solvers with good initial guesses and convergence guarantees. While implementing an FEM solver from scratch can be fairly tedious, several multipurpose computational libraries have appeared throughout the years, such as FEniCS (Alnæs *et al.*, 2015) or DUNE (Sander, 2020).

A clear disadvantage of this classical methodology is that it usually relies on a spatial discretization through, e.g. a spatial grid or a large polynomial basis, and thus, lets it suffer from the *curse of dimensionality*: in three space dimensions, it can already be difficult to employ, e.g. the FEM, see, e.g. the discussion in Bungartz & Griebel (2004). In filtering and optimal control, we are easily interested in PDEs occurring in hundreds or thousands of dimensions—here, classical methodology can rarely be employed. In addition, certain nonlinear and non-smooth PDEs are difficult to discretize with, e.g. finite elements due to behaviour that needs to be resolved on a very fine grid, general non-smooth behaviour, or singularities. Since FEM is a mesh-based solver, obtaining quickly converging solutions on certain irregular domains demands tailored approaches that are challenging to design and solve, see, e.g. Egger *et al.* (2014) for domains with re-entrant corners. On irregular domains, the *virtual element method* (Beirão da Veiga *et al.*, 2013) can be more appropriate than FEM.

In recent years, deep learning approaches have become a promising and popular methodology for the numerical solution of various PDEs. They have the potential to overcome some of the challenges that classical methods are facing. That is, neural networks have the advantage to not generally rely on a grid, similar to classical mesh free methods, such as Liu (2009). By leveraging automatic differentiation (Baydin *et al.*, 2018), they eliminate the need for discretization. Additionally, neural networks are able to represent more general functions than, e.g. an FEM basis, and they show evidence of beating the curse of dimensionality, see, e.g. Wojtowytsch & Weinan (2020); Hu *et al.* (2024). While the training of a neural network can become computationally demanding, especially when it consists of a non-convex optimization problem, it is very efficient when evaluating new data points once trained. In this emerging field of deep learning for approximating PDE solutions, the class of approaches closest to the classical methodologies is the one of function approximators (Tanyu *et al.*, 2023). They essentially model the solution as a deep neural network and train the network’s parameters to approximate the solution. Such approaches are e.g. the Deep Ritz method (Weinan & Bing, 2018) or the Deep Galerkin method (Sirignano & Spiliopoulos, 2018). A widely used and adapted method of this class are the so-called *physics-informed neural networks* (PINNs) (Raissi *et al.*, 2019) that will be the focus of this paper. Originally published in a two-part instalment (Raissi *et al.*, 2017a,b), Raissi *et al.* developed the PINNs approach (Raissi *et al.*, 2019)—we will refer to their approach as *vanilla PINNs* throughout the rest of this work. The basic idea behind PINNs is to minimize an energy functional that is the residual of the PDE and its initial and boundary conditions. The neural network itself models the solution function $u(t,x)$ given input variables $t$ and $x$ based on the underlying PDE. It has shown great success for many different types of PDEs (Mao *et al.*, 2020; Smith *et al.*, 2021) and has been extended to various specialized cases (Kharazmi *et al.*, 2019; Jagtap *et al.*, 2020; Yang *et al.*, 2021; Cuomo *et al.*, 2022).

While deep learning approaches for PDEs have gained a lot of traction in the last years and are being employed in increasingly more applications, they come with their set of challenges. In this work, we therefore systematically compare FEM and PINNs in a computational study. Before giving a complete outline of the following, we review recent works on PINNs.

### 1.1 PINNs: state of the art

Compared with FEM, the theoretical groundwork for PINNs is rather sparse. The first work on convergence results with respect to the number of training points is given by Shin *et al.* (Shin *et al.*, 2020). For linear second-order elliptic and parabolic PDEs, they prove strong convergence in $C^{0}$ for i.i.d. sampled training data. Mishra *et al.* (Mishra & Molinaro, 2022) have in turn developed upper bounds on the generalization error of PINNs given some stability assumptions on the PDE. Focusing on a specific PDE, Ryck *et al.* (De Ryck *et al.*, 2024) have investigated incompressible Navier–Stokes equations and provided an upper bound on the total error. However, their considerations are limited to the case of networks with two hidden layers and $\tanh $ activation functions. Despite the remaining need for more extensive theoretical work, PINNs have been extended into many different directions. Jagtap *et al.* (Jagtap *et al.*, 2020) develop conservative PINNs (cPINNs) to incorporate complex non-regular geometries by decomposing a spatial domain into independent parts and train separate PINNs for each. This work has been generalized to the extended PINNs (XPINNs) (Jagtap & Karniadakis, 2020) to allow space–time domain decomposition that can be applied to any type of PDE and enables parallelization in training. Another domain decomposition method for PINNs are the hp-VPINNs (Kharazmi *et al.*, 2021). This work is based on the variational PINNs (Kharazmi *et al.*, 2019) that take inspiration from classical approaches for solving PDEs. VPINNs form the loss functional based on the variational form of the PDE with Legendre polynomials as test functions, thus, allowing, e.g. for certain non-smoothnesses in PDEs and also for more efficient training. Bayesian PINNs (Yang *et al.*, 2021) for noisy data employ a Bayesian neural network. Finite basis PINNs (Moseley *et al.*, 2023) combine the PINN-idea with FEM, aiming to reduce the spectral bias in PINNs (Rahaman *et al.*, 2019). Noteworthy is also the work (Fabiani *et al.*, 2021), which uses a neural network with random weights to construct a basis on which PDEs can be solved efficiently. Interesting applications of PINNs appear in fluid dynamics (Mao *et al.*, 2020), electromagnetism (Kovacs *et al.*, 2022), elastic material deformation (Li *et al.*, 2021) and seismology (Smith *et al.*, 2021). For a more extensive review of PINNs, its applications and extended forms, we refer to the survey by Cuomo *et al.* (Cuomo *et al.*, 2022). Some shortcomings of PINNs are documented by Krishnapriyan *et al.* (Krishnapriyan *et al.*, 2021), who find that PINNs struggle to learn relevant physics in more challenging PDE regimes. However, they simultaneously present ideas to address these issues. Other shortcomings of PINNs in computational fluid dynamics were documented by Chuang & Barba (2022).

PINNs were created with physical application in mind. Due to their flexibility, the main building block can be kept the same across many physical problems with smaller adjustments to the architecture. Implementation of the framework has also become more approachable since the release of dedicated software packages such as DeepXDE (Lu *et al.*, 2021b), NVIDIA Modulus (previously SimNet) (Hennigh *et al.*, 2021) or NeuroDiffEq (Chen *et al.*, 2020).

### 1.2 Contributions and outline

As mentioned above, the main goal of this work is a systematic comparison of PINNs and FEM for the solution of PDEs. Indeed, we consider

- the elliptic Poisson equation in one, two and three space-dimensions,
- the parabolic Allen–Cahn equation in one space-dimension and
- the hyperbolic semilinear Schrödinger equation in one and two space dimensions.

We choose these model problems to cover a large range of classes of PDEs. We compare PINNs and FEM in terms of solution time, evaluation time and accuracy. We employ different finite element bases, as well as a multitude of network architectures. Several improvements over the vanilla PINNs have been discussed in the literature. As those are either still fundamentally based on the same idea or rather represent a mix of a classical approach and PINNs, we focus on vanilla PINNs in the following text. Throughout this work, we choose an experimental set-up that is supposed to mimic the way PINNs and FEM are used by practitioners, e.g. computational scientists and engineers, machine learners, mathematical modellers and so on.

This work is structured as follows. We discuss the PDEs, FEM and PINNs in Section 2. We introduce our method of comparison in Section 3, before presenting our computational results regarding Poisson, Allen–Cahn and semilinear Schrödinger equations in Sections 4, 5 and 6, respectively. We discuss our results and conclude the work in Section 7.

## 2. Mathematical background

In the following text, we first study a very general class of PDEs that we formally denote by 


(2.1) \begin{align*} \mathcal{A}u(x,t) = f(x,t) \quad x \in \varOmega, \,\, t \in [0,T].\end{align*}

Here, the function $u: \overline{\varOmega } \times [0,T] \to \mathbb{R}^{n}$ denotes the solution of the PDE, where $\varOmega \subset \mathbb{R}^{d}$ is open, bounded and connected and usually represents a spatial domain, whereas $[0,T]$ is the time interval. $\mathcal{A}$ denotes the differential operator acting on $u$ that can also be nonlinear and $f: \varOmega \times [0,T] \to \mathbb{R}^{n}$ is a source term. We denote the corresponding boundary conditions and the initial condition by 


(2.2) \begin{align*} \begin{split} \mathcal{B}u(x,t) &= g(x,t), \quad x \in \partial \varOmega, \,\, t \in [0,T] \\ u(x,0) &= h(x), \quad x \in \varOmega, \end{split}\end{align*}

respectively.

Throughout this work, we consider three different PDEs: the *Poisson equation*, the *Allen–Cahn equation* and the *semilinear Schrödinger equation*. We introduce those PDEs in the following text.

**Poisson equation.** The Poisson equation is a linear elliptic PDE of the form 


(2.3) \begin{align*} \varDelta u(x) = f(x), \qquad x \in \varOmega,\end{align*}

where $\varDelta = \sum _{i=1}^{d} \frac{\partial ^{2}}{\partial x_{i}^{2}} $ denotes the Laplacian and $f:\overline{\varOmega } \rightarrow \mathbb{R}$ is a source term. To be well defined, we need to equip it with boundary conditions, say, of *Dirichlet*-type: 


\begin{align*} u(x) = u_{\partial}(x), \qquad x \in \partial \varOmega, \end{align*}

*Neumann*-type: 


\begin{align*} \partial_{\vec{n}} u(x) = u_{\partial}(x) \qquad x \in \partial \varOmega, \end{align*}

or a combination of the two. In each case, we employ the function $u_{\partial }: \partial \varOmega \rightarrow \mathbb{R}$ to model the boundary behaviour. There are several results about the existence of strong and weak solutions of elliptic PDEs, see, e.g. Theorem 3 in Chapter 6.2 in Lawrence (2010). The Poisson equation models, for instance, the stationary heat distribution in a homogeneous object $\varOmega $; here, $f$ describes heat sources and sinks.

**Allen–Cahn equation.** The Allen–Cahn equation is a semilinear parabolic PDE of the form 


\begin{align*} & \frac{\partial u(t,x)}{\partial t}= \varepsilon\varDelta u(t,x) - \frac{2}{\varepsilon}u(t,x) (1-u(t,x)) (1-2u(t,x)), \qquad t \in [0, T],\, x \in \varOmega, \end{align*}

where $\varepsilon>0$ and which, of course, is considered together with appropriate boundary conditions and an initial condition. If $\varepsilon $ is sufficiently small, the solution of the Allen–Cahn equation approximately partitions the domain $\varOmega $ into patches where $u \approx 0$ and $u\approx 1$; in-between those patches—at the so-called diffuse interface—it is smooth. These patches can represent binary alloys (Allen & Cahn, 1972) or, e.g. different segments in an image with pixels in $\varOmega $ (Beneš *et al.*, 2004). The Allen–Cahn equation is also a relaxation of the mean-curvature flow to which it converges as $\varepsilon \downarrow 0$ (Feng & Prohl, 2003).

**Semilinear Schrödinger equation.** The semilinear Schrödinger equation is a complex-valued nonlinear hyperbolic PDE of the form 


\begin{align*} & \mathrm{i}\frac{\partial h(t,x)}{\partial t} = - \varDelta h(t,x) - h(t,x)|h(t,x)|^2, \qquad t \in [0, T], \,x \in \varOmega, \end{align*}

again subject to appropriate initial and boundary conditions. We have represented the semilinear Schrödinger equation above as it is usual in the literature. We present the PDE again splitting real and imaginary parts. We set $h(t,x) =: u_{R}(t,x) + \mathrm{i}u_{I}(t,x)$ and write 


\begin{align*} \frac{\partial u_{R}(t,x)}{\partial t} &= - \varDelta u_{I}(t,x) - (u_{R}^{2}(t,x) +u_{I}^{2}(t,x))u_{I}(t,x), \qquad t \in [0, T], \,x \in \varOmega,\\ \frac{\partial u_{I}(t,x)}{\partial t} &= \varDelta u_{R}(t,x) + (u_{R}^{2}(t,x) +u_{I}^{2}(t,x))u_{R}(t,x), \qquad t \in [0, T], \,x \in \varOmega. \end{align*}

We refer to Rozenman *et al.* (2020) and Strauss (1978) for an application of the semilinear Schrödinger equation in fluid dynamics and in nonlinear optics, respectively.

### 2.1 Finite element method

As mentioned before, FEM has been the gold standard for the spatial discretization of a huge class of PDEs. We now discuss the basics of FEM given the example of an elliptic PDE as in (2.3) with homogeneous Dirichlet boundary $u_{\partial } = 0$ and square integrable source $f \in \mathcal{L}^{2}(\varOmega )$. We mention non-stationary PDEs and other boundary conditions further below.

When solving an elliptic PDE with FEM, we aim to find a weak solution. That means, we try to find $u$ in an appropriate function space $U$ such that for all functions $v$ in an appropriate function space $V$, we have 


\begin{align*} & \int_\varOmega v(x) (\varDelta u(x) - f(x)) \mathrm{d}x = 0. \end{align*}

Applying integration by parts, we obtain the usual weak formulation of the Poisson equation: 


(2.4) \begin{align*}& \int_{\varOmega} \langle \nabla v(x), \nabla u(x)\rangle + v(x)f(x) \mathrm{d}x = 0.\end{align*}

An appropriate choice of function spaces $U$ and $V$ in this case is $U = V = H_{0}^{1}(\varOmega )$, the Sobolev space of square-integrable functions $\varOmega \rightarrow \mathbb{R}$ that are zero at the boundary $\partial \varOmega $ and that have a square-integrable weak derivative. In finite elements, we now replace $H_{0}^{1}(\varOmega )$ by a finite-dimensional subspace $H \subseteq U$ with basis $(\varphi _{i})_{i=1}^{N}$. On this finite-dimensional space, we can write the weak form (2.4) as 


(2.5) \begin{align*}& -\int_{\varOmega} \langle \nabla \varphi_{i}(x), \nabla \left( \sum_{j=1}^{N} a_{j}\varphi_{j}(x) \right)\rangle \mathrm{d}x = \int_{\varOmega} \varphi_{i}(x)f(x) \mathrm{d}x \qquad i=1,...,N,\end{align*}

which can be rewritten as a usual linear system $Ba = b$, with 


\begin{align*} & B = \left(-\int_\varOmega \langle \nabla \varphi_i(x), \nabla \varphi_j(x)\rangle \mathrm{d}x \right)_{i,j=1}^N, \qquad b = \left(\int_\varOmega \varphi_i(x)f(x) \mathrm{d}x \right)_{i=1}^N. \end{align*}

Now, we use $\sum _{j=1}^{N} a_{j}\varphi _{j}$ to approximate the solution $u$ of the PDE and obtain convergence to $u$ when appropriately choosing $(\varphi _{i})_{i=1}^{N}$, e.g. (Iserles, 2008, Theorem 9.3). While this approach allows for a large range of such bases, we usually speak only of finite elements when choosing specific locally supported, piecewise polynomial functions. As usual differential operators are local, this will usually lead to $B$ having a favourable, sparse structure.

In the discussion above, we consider homogeneous Dirichlet boundary conditions $u_{\partial } = 0$. Inhomogeneous boundary conditions, in which $u_{\partial }$ is not constantly $0$, can be achieved by first finding a function that satisfies the boundary conditions and then solving an auxiliary homogeneous problem with $u_{\partial } = 0$. The weak formulation for Neumann conditions adds the additional term $\int _{\partial \varOmega } v u_{\partial } \mathrm{d}x$ to the bilinear form and is solved on a different function space $U = V = H^{1}(\varOmega )$. Indeed, this is the Sobolev space of square-integrable functions with square-integrable weak derivatives. Mixed boundary conditions can be enforced in a similar way.

The time-domain of a non-stationary PDE can also be discretized with finite elements, see, e.g. Hulbert & Hughes (1990). We, however, consider the *method of lines* using finite elements in space and usual integrators for initial value problems in time, see, e.g. Schiesser & Griffiths (2009). To represent Allen–Cahn and semilinear Schrödinger at the same time, we consider some semilinear PDE of the form


\begin{align*} & \frac{\partial u(t,x)}{\partial t} = \varDelta u(t,x) + F(u(t,x)), \quad u(0,x) = u_0(x) \qquad t\in[0,T], \,x \in \varOmega, \end{align*}

which is also subject to boundary conditions. Due to the stiffness of the heat equation, we are required to use implicit techniques, such as the implicit Euler method. In a semi-discrete form, we can write the update step as 


\begin{align*} &u_{k+1} = u_k + \delta t \varDelta u_{k+1} + \delta t F(u_{k+1}) \qquad k = 0, 1, \ldots,\end{align*}

where $\delta t>0$ denotes the size of the time steps and $u_{k}(x) = u(t_{k},x)$ for $k= 0,1,\ldots $. In the weak formulation, we obtain 


\begin{align*} &\int_{\varOmega} v u_{k+1} \mathrm{d}x = \int_{\varOmega} v u_k - \delta t\langle \nabla v, \nabla u_{k+1}\rangle + \delta t v F(u_{k+1}) \mathrm{d}x \qquad v \in V, k = 0, 1, \ldots\end{align*}

and can now again use a finite element discretization in space to obtain an approximation of $u_{k+1}$ represented through a finite-dimensional equation, similar to (2.5), with the $(a_{j})_{j=1}^{N}$ now depending on time. In the case of Allen–Cahn and semilinear Schrödinger, this equation is nonlinear and requires us to repeatedly employ a Newton’s method.

We can prevent the additional cost of a Newton-solve and usually still obtain a stable discretization, by employing a semi-implicit strategy: linear parts of the PDE are solved with an implicit discretization, while nonlinear parts are discretized explicitly. In our setting, we obtain 


\begin{align*} &u_{k+1} = u_k + \delta t \varDelta u_{k+1} + \delta t F(u_{k}) \qquad k = 0, 1, \ldots\end{align*}

which requires us to solve the following weak problem: 


\begin{align*} &\int_{\varOmega} v u_{k+1}+ \delta t \langle \nabla v, \nabla u_{k+1}\rangle \mathrm{d}x = \int_{\varOmega} v u_k + \delta tv F(u_{k}) \mathrm{d}x \qquad v \in V,\, k = 0, 1, \ldots\end{align*}

which is fully linear.

For more details on FEM, we refer to, e.g. the book by Braess (Braess, 2007) or the classical references Courant (Courant, 1943) and Hrennikoff (Hrennikoff, 2021). Integrators for initial value problems, as well as evolution equations, are thoroughly considered by Iserles (Iserles, 2008). Semi-implicit schemes are thoroughly discussed by Koto (2008) and appear, for instance, in the work by Bertozzi and Schönlieb (Bertozzi & Schönlieb, 2010).

### 2.2 Physics-informed neural networks

The aim of PINNs is to approximate PDE solutions using a deep neural network. They use the powerful tool that is automatic differentiation and therefore do not rely on discretization of the space-time domain but rather on random sampling of the domain.

Let us consider a PDE of the form (2.1) with suitable boundary and initial conditions (2.2). The vanilla PINNs approach, as introduced by Raissi *et al.* (Raissi *et al.*, 2019), uses a fully connected neural network with $N_{l}$ hidden layers and $N_{e}(l)$ neurons per layer $l$. Inputs of the network are the independent variables $(x,t)$ sampled in the domain $\varOmega \times [0,T]$. The neural network acts as the function approximator $u_{\theta }(x,t)$ of the PDE solution, with $\theta $ the network weights that are optimized during training. Although FEM approximates the PDE solution on a space spanned by a basis of finite elements, PINNs approximate PDEs on the so-called Barron spaces—those were studied in Ma & Lei (2022). The PDE is integrated as a soft constraint in the optimization. That is, the network is trained with the PDE residual, as well as boundary and initial condition residuals as the loss functional: 


(2.6) \begin{align*}Loss(\theta) = &\frac{1}{N_f} \sum_{i = 1}^{N_f} \lVert \mathcal{A}u_{\theta}(x^i_f,t^i_f) - f(x^i_f,t^i_f)) \rVert^2 \end{align*}

(2.7) \begin{align*} &+ \frac{1}{N_{g}} \sum_{j = 1}^{N_{g}} \lVert \mathcal{B}u_{\theta}(x^{j}_{g},t^{j}_{g}) - g(x^{j}_{g},t^{j}_{g}) \rVert^{2} + \frac{1}{N_{h}} \sum_{k = 1}^{N_{h}} \lVert u_{\theta}(x^{k}_{h},0) - h(x^{k}_{h}) \rVert^{2}.\end{align*}

Here, $N_{f}$ is the number of collocation points $(x_{f}^{i}, t_{f}^{i}) \in \varOmega \times [0,T]$ for $i = 1, \dots , N_{f}$ sampled for the PDE residual in the loss. Similarly, $(x_{g}^{j},t_{g}^{j}) \in \partial \varOmega \times [0,T]$ for $j = 1, \dots , N_{g}$ denote the training points on the boundary and $x_{k}^{h} \in \varOmega $ for $k = 1, \dots , N_{h}$ the training data for the initial condition. Additionally, we need data $g(x^{j}_{g},t^{j}_{g})$ for the boundary and $h(x^{k}_{h})$ initial conditions. This can be measured data and does not need to be represented as an analytic function. PINNs is therefore able to incorporate measurements and leverage data-driven information. In the following text, we will only be using the 2-norm for the loss; however, this can be amended depending on the problem. The choice of $N_{f}, N_{g}$ and $N_{h}$ is dependent on the domain and complexity of the problem determined heuristically in our case. We follow Raissi *et al.* (2019) in using Latin Hybercube Sampling (Stein, 1987), a quasi-random approach for space filling sampling, to obtain the collocation points for training. In our experiments, we re-sample the collocation points in every epoch to get a better coverage of the sampling domain; see also Jin *et al.* (2023). As mentioned above, the differential operator $\mathcal{A}$ and any derivatives in the boundary condition operator $\mathcal{B}$ are evaluated using automatic differentiation (Baydin *et al.*, 2018). In contrast to numerical methods such as finite differences, automatic differentiation uses the chain rule to backpropagate through the network and evaluate the derivative. It is therefore not dependent on a grid or mesh that discretizes the domain. In turn, the sampling method becomes more important. Automatic differentiation gives rise to a significant advantage of PINNs towards other classical numerical methods for solving PDEs; it does not need to resolve derivatives on small grids, as in finite differences, and scales well in higher dimensions. In PINNs, the design of the neural network architecture, i.e. the type of network structure, the number of hidden layers and the number of nodes per layer, is flexible and can be adjusted based on the PDE complexity. In the following text, we will use fully connected feed-forward dense neural network. We denote the size and numbers of nodes per layers as $[N_{e}(1),N_{e}(2),...,N_{e}(l)]$. That is, $[5,5,1]$ e.g. represents a neural network with two hidden layers and five nodes per layer. The last entry 1 represents the size of the output layer. Lastly, the loss function is typically minimized using first the Adam optimizer (Kingma & Ba, 2015) for a coarser optimization and then the second-order quasi-Newton optimizer L-BFGS (Liu & Nocedal, 1989) which we found improved the accuracy.

## 3. Method of comparison

In this section, we give an overview of the experimental design and the measures that we consider to compare FEM and PINNs. The main features that we investigate are the time to solve the PDEs and the accuracy of the results. We therefore ask the questions of which methodology is computationally faster, more accurate and most efficient. We investigate different types of PDEs with varying complexity and dimensionality to cover a broad spectrum of PDEs and examine if computational speed and accuracy of the two approaches change based on the PDE type.

### 3.1 Experimental set-up

We now discuss the experimental set-up of our computational study. We first discuss ground truth solutions, and then describe the set-up for FEM and PINNs and the metrics with which we compare them.

Indeed, for a systematic comparison of FEM and PINNs, we need a ground truth solution of the PDEs to compare the solution approximations and to evaluate their accuracy. The first step is therefore to determine these ground truth solutions. For the Poisson equations, we have analytical solutions that we can use to determine the accuracy of the approximation approaches. However, neither the Allen–Cahn equation nor the semilinear Schrödinger equation have analytical solutions. Instead, we use FEM on a very fine mesh to compute a very accurate reference solution that we will use as the ground truth; time-stepping is performed using the implicit Euler method. One of the advantages of FEM is that we have extensive theoretical foundations for the convergence for large classes of PDEs, including the Allen–Cahn equation, e.g. Feng & Hai-jun (2005) and the semilinear Schrödinger equation, e.g. Shi *et al.* (2016). Thus, a finely meshed finite element solution can therefore guarantee accuracy up to a small error.

Let us now discuss the details and specifications for FEM that we consider in the comparison. As mentioned before, finer meshes will lead to higher accuracy albeit slower computation time. We therefore solve the PDEs for different mesh sizes to see the relationship between computation time and accuracy based on the FEM design. For time-dependent PDEs, the Allen–Cahn and the semilinear Schrödinger equations, we choose a semi-implicit strategy for discretization as detailed in Subsection 2.1. All FEM solution approximations are implemented using the Python toolbox FEniCS (version 2019.1.0) (Logg *et al.*, 2012; Alnæs *et al.*, 2015).

In the case of PINNs, we closely follow the vanilla approach as described in Raissi *et al.* (Raissi *et al.*, 2019). That is, we design the loss functional (2.6) and choose weights in the Allen–Cahn loss heuristically, elsewhere they are equal to 1. We use the Adam optimizer (Kingma & Ba, 2015) in the first instance and L-BFGS (Liu & Nocedal, 1989) to refine the optimization. The combination of the two optimizers allows for a fast coarse optimization followed by a computationally expensive refinement. We observed this to improve accuracy. The learning rate is heuristically chosen for optimal results in each PDE. All derivatives in the loss functional are computed using automatic differentiation. The collocation points that make up the input to the neural network are newly sampled for each epoch using Latin Hybercube sampling (Stein, 1987) in the Adam optimization. This way, we are able to cover more of the sampling space and improve generalizability of the trained network. L-BFGS does not allow for re-sampling between iterations and we therefore only sample the collocation points once before the optimization again using Latin Hybercube sampling. The number of collocation points were chosen heuristically and we do not use mini-batching in Adam. PINNs allow for a flexible design of the neural network architecture based on the underlying PDE. We therefore run the network training on architectures of different sizes, i.e. with varying numbers of layers and nodes. The code for PINN training and evaluation was written with the Python neural network library jax (Bradbury *et al.*, 2018) and is available on github: https://github.com/TamaraGrossmann/FEM-vs-PINNs.

We consider three main features for comparison. That is, we evaluate the FEM and PINNs approaches based on their solution time, evaluation time and accuracy. The solution time refers to the time it takes for each method to approximate the general solution. We differentiate between solution and evaluation time due to the way FEM and PINNs approximate solutions differently. FEM, typically, approximates the PDE solution on a fixed mesh. The solution can be approximated beyond the mesh by evaluation of the basis functions and/or interpolation—we always use linear interpolation in time. On the other hand, for PINNs a neural network is trained on a set of collocation points. The trained network can then be evaluated at any point in the domain. While the training time of PINNs can be rather slow, one of the advantages of neural networks is that once trained the evaluation on new input data require only a forward evaluation of the neural network, which is very fast. We therefore consider both times. For FEM this means solution time refers to assembling the matrix and solving the weak form of the PDE on a fixed mesh. FEM is run on a CPU. Considering PINNs, the solution time refers to the training time of the neural network which is run on a GPU. In turn, the evaluation time in FEM is measured as the time to interpolate the solution on a different mesh. In PINNs, the evaluation time is the time to evaluate the trained neural network on a new set of collocation points. We measure the accuracy of both methods on the same mesh in comparison with the ground truth solutions. For the Allen–Cahn and Schrödinger equations the evaluation mesh is chosen as the fine mesh on which the ground truth solution was derived. The measure for accuracy is the $\ell ^{2}$ relative error. All experiments were run on two machines with identical specifications: 12 CPU cores (Intel® Xeon® CPU E5-2643 v3 @ 3.40GHz) and a single Nvidia Quadro P6000 GPU with a base clock speed of 1506MHz. The codes were run 10 times and the reported solution and evaluation times are the average over the 10 recorded times.

It is not easy to find an experimental set-up that is appropriate to compare PINNs and FEM. With the set-up explained above, we aim to compare PINNs and FEM in the way they are intended to use by applicants. The FEM can sometimes be significantly sped-up using a GPU acceleration, see, e.g. Kiran *et al.* (2023). However, the standard mode of operation for the FEM appears to be based on CPUs. Similarly, modern automatic differentiation libraries are based on GPU-accelerated backpropagation, see, e.g. Sierra-Canto *et al.* (2010). We would assume a much slower training time of the PINNs, if we applied the backpropagation purely CPU-based. Thus, the way of comparison we use is appropriate in the sense that it represents the usual way in which PINNs and FEM is used by applicants.

## 4. Approximating the Poisson equation

Let us first investigate the Poisson equation in one, two and three space-dimensions. Each of the equations we consider has an analytical solution that we can use to evaluate the accuracy of the FEM and PINN approximation. Comparing the same type of PDE in different dimensions also allows us to draw conclusions about cost and accuracy effects due to the dimensionality of the PDE.

### 4.1 1D

For the one-dimensional case, we are examining the Poisson equation with a right-hand side $f(x)$ as detailed below on a unit interval and employ Dirichlet boundary conditions: 


(4.1) \begin{align*} \begin{split} \varDelta u(x) &= (4x^{3} - 6x) \exp{(-x^{2})}, \quad x \in (0,1), \\ u(0) &= 0,\\ u(1) &= \exp(-1). \end{split}\end{align*}

This PDE has the analytical solution 


\begin{align*} u_{\mathrm{true}}(x) = x\exp{(-x^{2})}, \qquad x \in [0,1]. \end{align*}

**Fig. 1.**
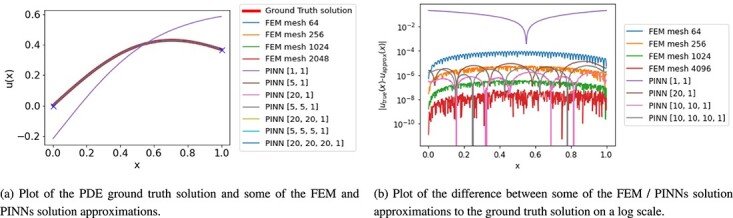
Plot for 1D Poisson equation solution.

A visualization of the solution to the 1D Poisson equation is shown in Fig. 1(a).

#### 4.1.1 FEM

As introduced in Section 2.1, the first step in solving (4.1) with FEM is deriving the weak formulation of the PDE, which we have done for Poisson equation already in (2.4). Here, of course $f(x) = (4x^{3} - 6x) \exp{(-x^{2})}$. Next, we need to define the finite element mesh. We choose a regular mesh on the domain $[0,1]$ with varying numbers of cells $n \in \{64,128,256,512,1024,2048,4096\}$. Of course, a higher number of cells corresponds to a finer grid on which the PDE is solved and subsequently leads to a more accurate, but also computationally more costly solution. The finite elements we are using are standard linear Lagrange elements, i.e. piecewise linear hat functions ($P_{1}$). Subsequently, we solve the variational problem in (2.4) with the Dirichlet boundary conditions specified in (4.1) using the conjugate gradient method with incomplete LU factorization as a preconditioner. All specifications are implemented using the python toolbox FEniCS (Logg *et al.*, 2012; Alnæs *et al.*, 2015) to compute an approximate PDE solution.

#### 4.1.2 PINNs

For solving the 1D Poisson equation using PINNs, there are three design parameters that we need to specify before training. The first step is choosing a loss functional. Following the vanilla PINNs approach, we evaluate the goodness of the solution using the discretized $\ell ^{2}$-energy or mean squared error over the PDE, boundary and initial conditions. In particular, we define the loss function as 


\begin{align*} \mathrm{Loss}(\theta) = &\frac{1}{N} \sum_{i = 1}^{N} \lVert \varDelta u_{\theta}(x_{i}) - (4x_{i}^{3} - 6x_{i}) \exp{(-x_{i}^{2})} \rVert^{2}_{2} + \lVert u_{\theta}(0) \rVert^{2}_{2} + \lVert u_{\theta}(1) - \exp(-1) \rVert^{2}_{2}, \end{align*}

with $u_{\theta }$ the neural network, $\theta $ the trained weights and $N = 256$ the number of collocation points $x_{i}$ sampled in each epoch using latin hybercube sampling. The second design parameter is the neural network architecture, i.e. the type of neural network, the activation function and the number of hidden layers and nodes. For the 1D Poisson case, we train feed-forward dense neural networks with $\tanh $ as the activation function. We compare the results on architectures of different sizes. The network architectures we consider for 1D Poisson are $[1,1], [2,1], [5,1], [10,1], [20,1], [40,1], [5,5,1], [10,10,1],$  $[20,20,1], [40,40,1], [5,5,5,1], [10,10,10,1], [20,20,20,1]$ and $[40,40,40,1]$. The loss function is minimized for each network architecture using the Adam optimizer for $15,000$ epochs with a learning rate of $10^{-4}$ in the first instance. Additionally, we refine the optimization using L-BFGS.

#### 4.1.3 Results

The resulting solution approximations of the 1D Poisson equation using FEM and PINNs are compared with the analytical solution on a mesh in $[0,1]$ with 512 mesh points. The ground truth solution of the 1D Poisson equation in (4.1) and its approximations are displayed in Fig. 1(a). Likewise, the difference from the approximate solutions to the GT solution is shown in Fig. 1(b) for all mesh sizes of FEM and architectures in PINNs. One of the architectures in the PINNs approximation renders results with large relative error: the PINN with a single hidden layer and one node is not even able to learn the solution in a way in which the boundary conditions are satisfied. However, all other solution approximations differ from the ground truth only marginally.

**Fig. 2.**
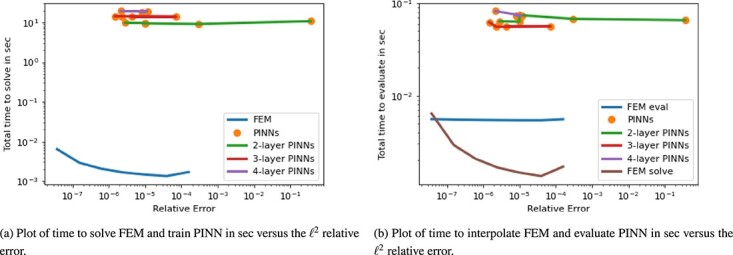
Plot for 1D Poisson equation of time in sec versus the $\ell ^{2}$ relative error.

Let us compare the time it takes to solve or rather approximate the PDE using FEM and PINNs to the relative error produced on a new set of grid points. For FEM the solution time is the time to solve the linear systems and for PINNs we consider the time to train the neural network. The results are shown in Fig. 2(a). We can clearly show that overall FEM is faster and more accurate in their solution approximation. While there are some PINN architectures that are able to achieve similar or even lower relative errors than the coarser FEM approximations, their training time is two to three orders of magnitude higher than in FEM. Considering the evaluation time, i.e. the time to interpolate the FEM solution on a different mesh and evaluate the trained PINN on a test set, we can see a similar relationship; see Fig. 2(b). FEM solution approximations are overall faster and more accurate. Finally, we consider the relationship between the number of layers and the solution time and relative error. We observe that the time to train a PINN is similar relative to the number of layers. However, the accuracy of each network is related to the number of nodes in the layers. That is, we cannot show that networks with more layers generally achieve better results in 1D Poisson.

### 4.2 2D

Let us now investigate a two-dimensional Poisson equations given by


(4.2) \begin{align*} \begin{split} \varDelta u(x,y) = 2& (x^{4} (3 y - 2) + x^{3} (4 - 6 y) + x^{2} (6 y^{3} - 12 y^{2} + 9 y - 2) \\ & - 6 x (y - 1)^{2} y + (y - 1)^{2} y) \qquad \qquad \qquad \qquad (x,y) \in (0,1)^{2} \end{split} \end{align*}

(4.3) \begin{align*} \begin{split} \partial_{\vec{n}} u(0,y) &= 0 \quad y \in [0,1]\\ \partial_{\vec{n}} u(1,y) &= 0 \quad y \in [0,1]\\ u(x,0) &= 0 \quad x \in [0,1]\\ \partial_{\vec{n}} u(x,1) &= 0 \quad x \in [0,1] \end{split}\qquad\qquad\qquad\qquad\qquad\qquad\qquad\qquad\qquad \qquad\end{align*}

We can solve the PDE (4.2) with mixed boundary conditions analytically: 


\begin{align*} u_{\mathrm{true}}(x,y) = x^{2} (x-1)^{2} y (y-1)^{2}, \qquad (x,y) \in (0,1)^{2}. \end{align*}

**Fig. 3.**
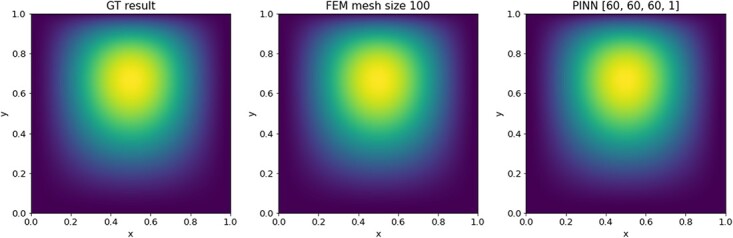
Comparison of the 2D Poisson ground truth solution to examples of the FEM and PINN approximations.

The solution is displayed in Fig. 3.

#### 4.2.1 FEM

For the 2D Poisson equation, we again refer to the weak formulation of the Poisson equation in (2.4) with $f(x,y) = 2 (x^{4} (3 y - 2) + x^{3} (4 - 6 y) + x^{2} (6 y^{3} - 12 y^{2} + 9 y - 2) - 6 x (y - 1)^{2} y + (y - 1)^{2} y)$. The finite element mesh is defined on the unit square $[0,1] \times [0,1]$; it contains $n \times n$ squares with $n \in \{100, 200,\ldots ,1000\}$. Each square on the mesh is divided into two triangles on which we define again piecewise linear finite elements ($P_{1}$). We solve the variational problem (2.4) with mixed boundary conditions given in (4.3) using the conjugate gradient method with incomplete LU factorization preconditioning. The method is implemented using FEniCS.

#### 4.2.2 PINNs

The loss functional for the PINN approximation is the $\ell ^{2}$-residual of the PDE (4.2) and its boundary conditions. For $u_{\theta }$ the neural network with weights $\theta $ that are to be trained, the loss reads: 


\begin{align*} \text{Loss}&(\theta) \\ = \, &\frac{1}{N_{f}} \sum_{i = 1}^{N_{f}} \lVert \varDelta u_{\theta}(x^{i}_{f},y^{i}_{f}) - 2 ((x^{i}_{f})^{4} (3 y^{i}_{f} - 2) + (x^{i}_{f})^{3} (4 - 6 y^{i}_{f}) + (x^{i}_{f})^{2} (6 (y^{i}_{f})^{3} - 12 (y^{i}_{f})^{2} + 9 y^{i}_{f} - 2) \rVert^{2}_{2} \\ &+ \frac{1}{N_{g}} \sum_{j = 1}^{N_{g}}\left(\left\lVert \partial_{\vec{n}}u_{\theta} (0,y^{j}_{g})\right\rVert^{2}_{2} + \left\lVert \partial_{\vec{n}} u_{\theta} (1,y^{j}_{g})\right\rVert^{2}_{2} + \left\lVert u_{\theta}(x^{j}_{g},0)\right\rVert^{2}_{2} + \left\lVert \partial_{\vec{n}} u_{\theta}(x^{j}_{g},1)\right\rVert^{2}_{2} \right). \end{align*}

The collocation points are sampled at every epoch using Latin Hybercube sampling with $N_{f} = 2000$ and $N_{g} = 250$. We train a feed-forward dense neural network with $\tanh $ activation function. We consider 11 different architectures for training, these are [20,1], [60,1], [20,20,1], [60,60,1], [20,20,20,1], [60,60,60,1], [20,20,20,20,1], [60,60,60,60,1], [20,20,20,20,20,1], [60,60,60,60,60,1] and [120,120,120,120,120,1]. Just like in 1D Poisson, we use the Adam optimizer for $20,000$ epochs with a learning rate of $10^{-3}$ to train the network. Subsequently, we refine the optimization using L-BFGS.

#### 4.2.3 Results

**Fig. 4.**
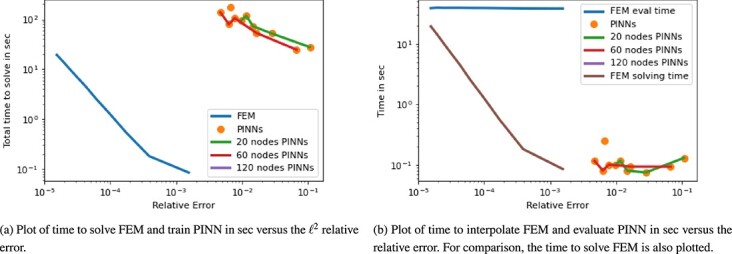
Plot for 2D Poisson equation of time in sec versus the $\ell ^{2}$ relative error.

An example of the resulting solution approximations on a mesh with $2000 \times 2000$ cells next to the ground truth solution is show in Fig. 3. The time versus accuracy plots for the 2D Poisson equation are displayed in Fig. 4(a) and Fig. 4(b). Considering the solution time, FEM clearly outperforms all PINN approximations both in accuracy and computation time. Using FEM to obtain the PDE solution is faster by one to three orders of magnitude. However, when we look at the evaluation times in Fig. 4(b), the relationship changes. That is, the time to evaluate a PINN is two to three orders of magnitude faster than the interpolation of FEM on a new mesh. We additionally plotted the FEM solution time in the same plot, and the PINN evaluation remains faster, however, by a smaller margin. Interestingly, the solution time of FEM is faster than the FEM evaluation time; this is possibly due to an inefficient interpolation code. Even though the evaluation of the trained neural network gives an improvement in time, the PINN approximations remain to have lower accuracy.

### 4.3 3D

For the three-dimensional Poisson equation, we choose a PDE on the unit cube with Dirichlet boundary conditions, as follows: 


\begin{align*} \varDelta u(x,y,z) &= -3 \pi^{2}\sin(\pi x)\sin(\pi y)\sin(\pi z), \quad (x,y,z) \in (0,1)^{3}, \\ u(x,y,z) &= 0, \quad (x,y,z) \in \partial(0,1)^{3}. \end{align*}

**Fig. 5.**
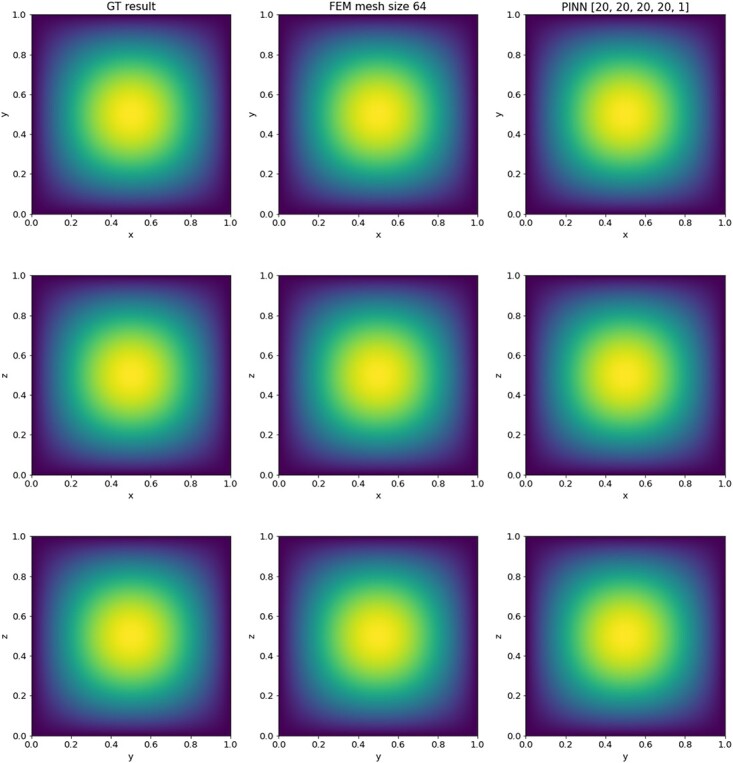
Comparison of the 3D Poisson ground truth solution slices at $x,y,z = 0.5$, respectively, to examples of the FEM and PINN approximations.

The analytical solution of this 3D Poisson equation is displayed in Fig. 5 and can be written as 


\begin{align*} u_{\mathrm{true}}(x,y,z) = \sin(\pi x)\sin(\pi y)\sin(\pi z), \quad (x,y,z) \in (0,1)^{3}. \end{align*}

#### 4.3.1 FEM

The weak formulation of the 3D Poisson equation is again given in (2.4) with $f(x,y,z) = -3 \pi ^{2}\sin (\pi x)\sin (\pi y)\sin (\pi z)$. The finite element mesh is defined on a unit cube $[0,1] \times [0,1] \times [0,1]$ consisting of $n \times n \times n$ cubes with $n \in \{16, 32, 64, 128\}$. We subdivide each cube into tetrahedrals and use again piecewise linear finite elements ($P_{1}$). The weak problem is solved using the conjugate gradient method and incomplete LU factorization as the preconditioner.

#### 4.3.2 PINNs

Similar to the one- and two-dimensional Poisson case, we design the loss functional as the PDE and boundary condition residual. That is, we define the loss as follows: 


\begin{align*} \text{Loss}(\theta) = \, &\frac{1}{N_{f}} \sum_{i = 1}^{N_{f}} \lVert \varDelta u_{\theta}(x^{i}_{f},y^{i}_{f},z^{i}_{f}) + 3 \pi^{2}\sin(\pi x^{i}_{f})\sin(\pi y^{i}_{f})\sin(\pi z^{i}_{f}) \rVert^{2}_{2} \\ &+ \frac{1}{N_{g}} \sum_{j = 1}^{N_{g}}\left(\lVert u_{\theta} (0,y^{j}_{g},z^{j}_{g})\rVert^{2}_{2} + \lVert u_{\theta} (x^{j}_{g},0,z^{j}_{g})\rVert^{2}_{2} + \lVert u_{\theta} (x^{j}_{g},y^{j}_{g},0)\rVert^{2}_{2}\right)\\ &+\frac{1}{N_{g}} \sum_{j = 1}^{N_{g}}\left(\lVert u_{\theta} (1,y^{j}_{g},z^{j}_{g})\rVert^{2}_{2} + \lVert u_{\theta} (x^{j}_{g},1,z^{j}_{g})\rVert^{2}_{2} + \lVert u_{\theta} (x^{j}_{g},y^{j}_{g},1)\rVert^{2}_{2}\right). \end{align*}

Here, $u_{\theta }$ denotes the neural network with $\theta $ the training parameters. We sample $N_{f} = 1000$ collocation points in the domain and $N_{g} = 100$ on the boundary. The neural network is again a feed-forward dense neural network with $\tanh $ activation function. We consider eight different architectures to train with the following layer and node specifications: [20,20,1], [60,60,1], [20,20,20,1], [60,60,60,1], [20,20,20,20,1], [60,60,60,60,1], [20,20,20,20,20,1] and [60,60,60,60,60,1]. The Adam optimizer with learning rate $10^{-3}$ is used for 20,000 epochs before employing the L-BFGS optimizer.

#### 4.3.3 Results

**Fig. 6.**
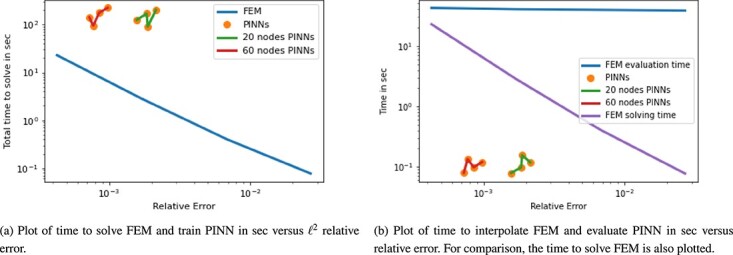
Plot for 3D Poisson equation of time in sec versus $\ell ^{2}$ relative error.

The PDE is evaluated on a mesh of $150 \times 150 \times 150$ grid points and example results are shown in Fig. 5. The time versus accuracy plots can be found in Fig. 6. As expected, the FEM results with slower computation times have lower relative errors as they are solved on finer meshes as shown in Fig. 6(a). While the PINN approximations are about one to three orders of magnitude slower in training time depending on the FEM mesh solution that we compare with, they are able to achieve equal and even higher accuracy in most cases. On the other hand, PINNs outperform FEM when considering the evaluation time as plotted in Fig. 6(b). The time to interpolate FEM on a new mesh is two to three orders of magnitude slower than the evaluation time of PINNs. Additionally, PINNs are able to achieve equal or, in the case of coarse FEM approximations, higher accuracy scores. If we also take the FEM solving time into account, we find again that this is faster than the evaluation time. However, it is slower than the PINNs evaluation. Therefore, a trained PINN has a lower computation time for the evaluation as compared with both the FEM solution and evaluation times.

## 5. Approximating the Allen–Cahn equation

To study the one dimensional Allen–Cahn equation, we consider the following PDE: 


(5.1) \begin{align*} \begin{split} \frac{\partial u(t,x)}{\partial t} &= \epsilon \varDelta u - \frac{2}{\epsilon} u(t,x) (1-u(t,x)) (1-2u(t,x)), \quad x \in \varOmega = [0,1], \,\, t \in [0,T],\\ u(t,0) &= u(t,1), \quad t \in [0,T],\\ u(0,x) &= \frac{1}{4}\left(\sin (2 \pi x) + \sin(16 \pi x) \right) + \frac{1}{2}, \quad x \in \varOmega, \end{split}\end{align*}

where $T = 0.05$ and $\epsilon = 0.01$. As mentioned in Section 2, we note that a smaller $\epsilon $ will yield close to piecewise constant solutions, whereas solutions with large $\epsilon $ will be overall more smooth. The $\epsilon $ chosen in this case is due to the inability of PINNs to approximate the Allen–Cahn solution with a smaller $\epsilon $. We have trained the neural network to solve Allen–Cahn with $\epsilon = 0.001$ for various network architectures and with activation functions such as softplus that are typically able to handle discontinuous solutions. However, the PINNs were not able to recover results close to the ground truth solution. We will discuss this case further below. For now, the results refer to $\epsilon = 0.01$.

### 5.0.1 FEM

As opposed to the Poisson equation, the Allen–Cahn equation has a time dependency that we need to consider in the discretization. As mentioned in Section 2.1, we use an implicit Euler strategy to approximate the solution. The weak formulation in a semi-discrete form of the 1D Allen–Cahn equation with homogeneous Dirichlet boundary $u_{\partial } = 0$ can be written as 


(5.2) \begin{align*} \begin{split} \int_{0}^{1} &(u_{k+1}(x) - u_{k}(x))v(x) \mathrm{d}x + \epsilon \int_{0}^{1} \langle \nabla u_{k+1}(x), \nabla v(x)\rangle \mathrm{d}x \\ &+ \frac{2}{\epsilon} \int_{0}^{1} u_{k+1}(x)(1-u_{k+1}(x))(1-2u_{k+1}(x))v(x) \mathrm{d}x = 0, \end{split}\end{align*}

for all test functions $v \in H^{1}_{0}([0,1])$. The time is discretized with distance of $dt = 10^{-3}$. For the finite element mesh in space, we choose an interval mesh on the domain $[0,1]$ with varying numbers of cells $n \in \{32,128,512,2048\}$. As for the 1D Poisson problem, we employ piecewise-linear finite element functions ($P_{1}$). The nonlinear variational problem (5.2) with periodic boundary conditions is solved using a Newton solver. The FEM approach was implemented using the Python toolbox FEniCS.

### 5.0.2 PINNs

We follow the general PINNs approach and design the loss based on the PDE residual, the boundary residual and the initial condition residual. Additionally, we introduce a weighting of the different terms in the loss functional. We found heuristically this weighting to render the best results. 


(5.3) \begin{align*} \begin{split} \text{Loss}(\theta) = \, &\frac{1}{N_{f}} \sum_{i = 1}^{N_{f}} \left\lVert \frac{\partial u_{\theta}(t^{i}_{f},x^{i}_{f})}{\partial t} - \epsilon \varDelta u_{\theta} (t^{i}_{f},x^{i}_{f}) + \frac{2}{\epsilon} u_{\theta}(t^{i}_{f},x^{i}_{f}) \big(1-u_{\theta}(t^{i}_{f},x^{i}_{f})\big) \big(1-2u_{\theta}(t^{i}_{f},x^{i}_{f})\big) \right\rVert^{2}_{2} \\ &+ \frac{1}{N_{g}} \sum_{k = 1}^{N_{g}}\lVert u_{\theta}(t^{j}_{g},0) - u_{\theta}(t^{j}_{g},1) \rVert^{2}_{2} \\ &+ \frac{1000}{N_{h}} \sum_{k = 1}^{N_{h}}\left\lVert u_{\theta}(0,x^{k}_{h}) - \frac{1}{4}\left(\sin (2 \pi x^{k}_{h}) + \sin(16 \pi x^{k}_{h}) \right) + \frac{1}{2}\right\rVert^{2}_{2}, \end{split}\end{align*}

with $u_{\theta }$ the neural network and $\theta $ the trained weights. We train the network on $N_{f} = 20,000$ collocation points $(t_{f}^{i},x_{f}^{i}) \in [0,0.05]\times [0,1]$ that are sampled using Latin Hybercubes. The training points for the boundary with $N_{g} = 250$ and for the initial condition with $N_{h} = 500$ are also sampled in each epoch with Latin Hybercube sampling. The network architecture is a feed-forward dense neural network with a $\tanh $ activation function. We consider architectures of the following 14 different sizes: [20,20,20,1], [100,100,100,1], [500,500,500,1], [20,20,20,20,1], [100,100,100,100,1], [500,500,500,500,1], [20,20,20,20,20,1], [100,100,100,100,100,1], [500,500,500,500,500,1], [20,20,20,20,20,20,1], [100,100,100,100,100,100,1], [500,500,500,500,500,500,1], [20,20,20,20,20,20,20,1] and [100,100,100,100,100,100,100,1]. For a network of size [500,500,500,500,500,500,500,1] we run out of memory on the GPU available to us. This constitutes the limitation of our training. For the optimization we first run the Adam optimizer with learning rate $10^{-4}$ for 7000 epochs over the initial loss alone. We have found the network struggling to learn the initial condition when the optimization is run on the full loss directly. After the 7000 epochs optimizing the initial loss, we run the Adam optimizer for $50,000$ epochs on the full loss function (5.3). Finally, we refine the optimization using L-BFGS.

### 5.0.3 Results

**Fig. 7.**
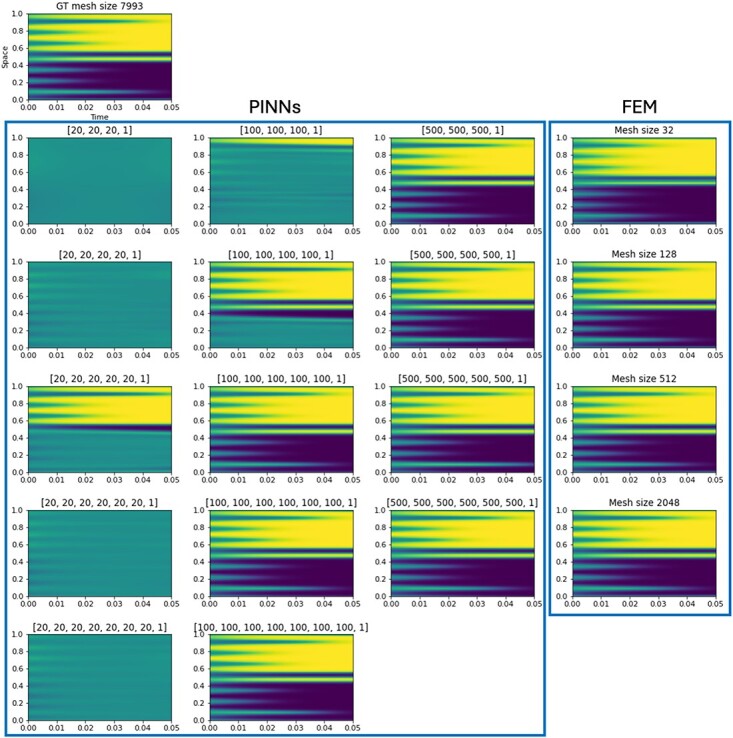
Comparison of the 1D Allen–Cahn solution approximated by FEM and PINNs.

The PDE approximations with FEM and PINNs are compared on the mesh of the ground truth solution spanning $[0,1]$ with 7993 mesh points and at a time discretization $dt =\frac{1}{3}\times 10^{-4}$ up to time $T = 0.05$. The fine-meshed FEM approximation for the ground truth solution was derived using implicit Euler. The FEM and the PINN approximations compared with the ground truth solution are displayed in Fig. 7 for the different mesh sizes and network architectures. FEM is able to recover the solution of the PDE at all mesh sizes. However, closer inspection of the result for a mesh of size $32$ shows slight errors along the diffusive interface. On the other hand, the ability of a PINN to approximate the PDE solution well is dependent on the architecture and the number of free parameters or weights that are to be determined. While all architectures with 20 nodes (cf. Fig. 7 column 1) are not able to recover the solution whatsoever, networks with 100 nodes per layer are able to be trained for the solution. We can clearly observe a progression based on the number of layers. Finally, neural networks that have 500 nodes per layer are all able to approximate the solution well.

**Fig. 8.**
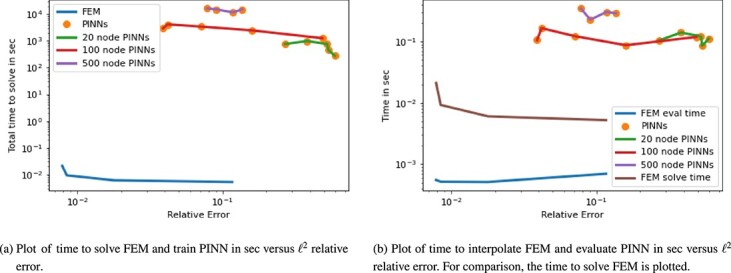
Plot for 1D Allen–Cahn equation of time in sec versus $\ell ^{2}$ relative error.

Let us compare the solution and evaluation time versus the accuracy for the FEM and PINN approximations as shown in Fig. 8. For the solution time, i.e. the time to solve the PDE using FEM and the time to train the neural network in PINN, FEM is five to six orders of magnitude faster than PINNs. This is mainly due to the size of the neural networks. Here, the complexity of the PDE requires larger architectures to be able to capture the solution. Some of the PINN architectures are then able though to achieve similar relative errors to FEM. This is especially true for networks with 100 or 500 nodes per layer as also seen in Fig. 7. The evaluation time of the PINN is plotted against the evaluation time of FEM and the solution time of FEM in Fig. 8(b). The calculation time advantage of FEM evaluation drops to about one order of magnitude as compared with the solution time. While it is to be expected that the evaluation of a neural network is much faster then the training, FEM is still faster in both solution and evaluation time.

**Fig. 9.**
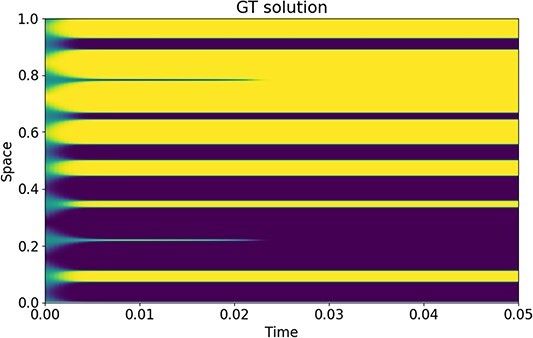
FEM solution of the 1D Allen–Cahn equation for $\epsilon = 0.001$ derived on a mesh with 7993 cells.

Compared with the other PDEs that we are considering in this study, the Allen–Cahn equation needs slight modification, i.e. weighting of the loss and pre-training on only part of the loss functional. The need for modification is due to the difficulty we have found the network to have in learning the PDE solution. In addition, we should note that we have also attempted to train a PINN for Allen–Cahn (5.1) with $\epsilon =0.001$. The resulting solution—shown in Fig. 9—becomes close to binary after a certain amount of time. We had discussed this effect in Section 2. This renders very large gradients in the solutions. We trained PINNs with different activation functions such as softplus or ReLU that are typically able to handle discontinuities. However, all results were insufficient to be considered an approximation. This speaks to the assumption that PINNs in the vanilla form are not well equipped to handle discontinuous solutions; this may also be due to PINNs solving the strong PDE, rather than a weak form. Variations of the vanilla PINNs approach might be able to obtain satisfactory approximations. However, as we are only considering the vanilla approach, this goes beyond the scope of the paper. We make a note that FEM is able to approximate Allen–Cahn with $\epsilon =0.001$ albeit we should anticipate the use of a finer mesh to accurate represent the diffuse interface.

## 6. Approximating the semilinear Schrödinger equation

Finally, we investigate the semilinear Schrödinger equation in one and two space-dimensions. The specifications of the one-dimensional case are taken from the original PINNs paper of Raissi *et al.* (Raissi *et al.*, 2019). Note that the semilinear Schrödinger equation has a complex-valued solution; further increasing the difficulty of its approximation.

### 6.1 1D

In the one-dimensional case, we consider the semilinear Schrödinger equation with periodic boundary conditions, as follows: 


\begin{align*} \mathrm{i}\frac{\partial h(t,x)}{\partial t} &= -0.5 \varDelta h(t,x) - \lvert h(t,x) \rvert^{2} h(t,x) &x \in [-5,5], \,\, t \in [0,\pi /2],\\[-3pt] h(0,x) &= 2 \text{sech}(x), &x \in [-5,5],\\[-3pt] h(t,-5) &= h(t,5), &t \in [0,\pi /2],\\[-3pt] \frac{\partial h(t,-5)}{\partial x} &= \frac{\partial h(t,5)}{\partial x}, &t \in [0,\pi /2]. \end{align*}

**Fig. 10.**
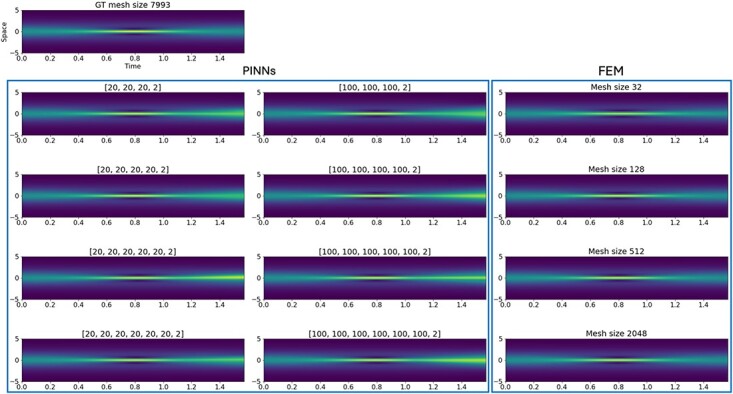
Comparison of the 1D semilinear Schrödinger solution $\lvert h(t,x) \rvert = \sqrt{u_{R}^{2}(t,x) + u_{I}^{2}(t,x)}$ approximated by FEM and PINNs of different mesh and architecture sizes.

We note that the identical problem had also been considered by Raissi *et al.* (2019). As $h(t,x)$ is a complex valued function, the semilinear Schrödinger equation is solved for $h(t,x) =: u_{R}(t,x) + \mathrm{i}u_{I}(t,x)$, with $u_{R}(t,x)$ the real part and $u_{I}(t,x)$ the imaginary part. Results are visualized for $\lvert h(t,x) \rvert = \sqrt{u_{R}^{2}(t,x) + u_{I}^{2}(t,x)}$ in Fig. 10.

#### 6.1.1 FEM

Similar to the 1D Allen–Cahn equation that we considered, we use a semi-implicit Euler strategy to approximate the 1D Schrödinger equation in time. We can then write the weak formulation for the real and imaginary parts of the PDE, assuming Dirichlet $u_{\partial } = 0$ boundaries, separately as 


\begin{align*} \int_{-5}^{5} (u^{I}_{k+1}(x) - u^{I}_{k}(x))v^{R}(x) \mathrm{d}x - 0.5 \int_{-5}^{5} \langle \nabla u^{R}_{k+1}(x), \nabla v^{R}(x)\rangle \mathrm{d}x - \lvert h_{k}(x) \rvert^{2} \int_{-5}^{5} u^{R}_{k+1}(x)v^{R}(x) \mathrm{d}x &= 0,\\[-3pt] \int_{-5}^{5} (u^{R}_{k+1}(x) - u^{R}_{k}(x))v^{I}(x) \mathrm{d}x + 0.5 \int_{-5}^{5} \langle \nabla u^{I}_{k+1}(x), \nabla v^{I}(x)\rangle \mathrm{d}x + \lvert h_{k}(x) \rvert^{2} \int_{-5}^{5} u^{I}_{k+1}(x)v^{I}(x) \mathrm{d}x &= 0, \end{align*}

for all test functions $v^{R}, v^{I} \in H_{0}^{1}(\varOmega )$. The time is discretized with $dt = 5\times 10^{-4}$ and we define the finite elements on an interval mesh in $[-5,5]$. We consider meshes with $n \in \{32,128,512,2048\}$ cells. We again employ piecewise linear finite element basis functions on those cells ($P_{1}$) and use the generalized minimal residual method (gmres) to solve the linear problems in the semi-implicit scheme.

#### 6.1.2 PINNs

Let us now define the loss functional used in the neural network approximation of the PDE solution. Again, we consider the residual of the PDE, inital condition and boundary conditions as follows: 


\begin{align*} \begin{split} \text{Loss}(\theta) = \, &\frac{1}{N_{f}} \sum_{i = 1}^{N_{f}} (\lVert \frac{\partial u^{I}_{\theta}(t^{i}_{f},x^{i}_{f})}{\partial t} - \epsilon \varDelta u^{R}_{\theta} (t^{i}_{f},x^{i}_{f}) - \lvert h_{\theta}(t^{i}_{f},x^{i}_{f}) \rvert^{2} u^{R}_{\theta}(t^{i}_{f},x^{i}_{f}) \rVert^{2}_{2} \\ &\qquad + \lVert \frac{\partial u^{R}_{\theta}(t^{i}_{f},x^{i}_{f})}{\partial t} + \epsilon \varDelta u^{I}_{\theta} (t^{i}_{f},x^{i}_{f}) + \lvert h_{\theta}(t^{i}_{f},x^{i}_{f}) \rvert^{2} u^{I}_{\theta}(t^{i}_{f},x^{i}_{f}) \rVert^{2}_{2} )\\ &+ \frac{1}{N_{g}} \sum_{k = 1}^{N_{g}}\left(\lVert u^{R}_{\theta}(t^{j}_{g},-5) - u^{R}_{\theta}(t^{j}_{g},5) \rVert^{2}_{2} + \lVert u^{I}_{\theta}(t^{j}_{g},-5) - u^{I}_{\theta}(t^{j}_{g},5) \rVert^{2}_{2} \right)\\ &+ \frac{1}{N_{g}} \sum_{k = 1}^{N_{g}}\left(\left\lVert \frac{\partial u^{R}_{\theta}(t^{j}_{g},-5)}{\partial x} - \frac{\partial u^{R}_{\theta}(t^{j}_{g},5)}{\partial x} \right\rVert^{2}_{2} + \left\lVert \frac{\partial u^{I}_{\theta}(t^{j}_{g},-5)}{\partial x} - \frac{\partial u^{I}_{\theta}(t^{j}_{g},5)}{\partial x} \right\rVert^{2}_{2} \right)\\ &+ \frac{1}{N_{h}} \sum_{k = 1}^{N_{h}}\left(\lVert u^{R}_{\theta}(0,x^{k}_{h}) - 2 \text{sech}(x^{k}_{h})\rVert^{2}_{2} + \lVert u^{I}_{\theta}(0,x^{k}_{h})\rVert^{2}_{2}\right), \end{split} \end{align*}

where $h_{\theta }(t,x) = [u^{R}_{\theta }(t,x),u^{I}_{\theta }(t,x)]$ is the neural network the produces the real and imaginary parts of the PDE solution of the network output with weights $\theta $. The network is trained on $N_{f} = 20,000$ collocation points $(t^{i}_{f},x^{i}_{f}) \in [0,\pi /2] \times [-5,5]$ and with $N_{g} = 50$ point on the boundary and $N_{h} = 50$ points for the initial condition using Latin Hybercube sampling. The network architecture is a feed-forward dense neural network with $\tanh $ activation function. In these specifications, we have followed the original vanilla PINNs paper (Raissi *et al.*, 2019). We investigate the performance of eight network architectures: [20,20,20,2], [100,100,100,2], [20,20,20,20,2], [100,100,100,100,2], [20,20,20,20,20,2], [100,100,100,100,100,2], [20,20,20,20,20,20,2] and [100,100,100,100,100,100,2]. We employ the Adam optimizer for $50,000$ epochs and a learning rate of $10^{-4}$. Afterwards, we use the L-BFGS optimizer to refine the training results.

#### 6.1.3 Results

**Fig. 11.**
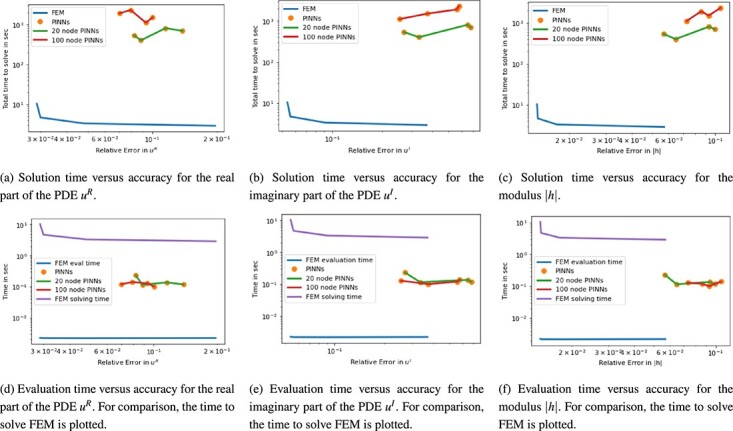
Plot for 1D Schrödinger equation of time in sec versus $\ell ^{2}$ relative error. Plots are split for the real and imaginary parts of the PDE as well as $\lvert h \rvert = \sqrt{u^{2}_{R} + u_{I}^{2}}$.

The results of the FEM and PINNs approximation are compared on the mesh of the ground truth solution that has a size of 7993 cells and a time discretization with $dt = \frac{1}{3}\times 10^{-4}$. The resulting approximations for $\lvert h(t,x) \rvert = \sqrt{u^{2}_{R}(t,x) + u_{I}^{2}(t,x)}$ are displayed in Fig. 10. Already visually, we notice that the PINN approximations become slightly less accurate for larger time instance than the FEM approximations. Quantitatively speaking, the time versus accuracy plots give a broader overview of the performance of each method. The plots are shown in Fig. 11. Focusing on the modulus $\lvert h \rvert $, FEM both has a lower solution time by two orders of magnitude and a lower relative error than any neural network approximation as shown in Fig. 11(c). Considering the evaluation time for both methods alone, FEM continues to outperform PINNs in time and accuracy. However, the PINN evaluation time is faster than the solution time of FEM. Nonetheless, FEM remains to produce results with higher accuracy for $|h|$. The approximation results are slightly less accurate for $u_{I}$. Also there, however, the FEM solution reaches the same or a better accuracy than the PINNs solution at a much faster pace, as we see in Fig. 11(b) and 11(e).

### 6.2 2D

Let us finally move to the two-dimensional semilinear Schrödinger equation. We consider periodic boundary conditions and define the initial condition as follows: 


\begin{align*} \mathrm{i}\frac{\partial h(t,x,y)}{\partial t} &= -0.5 \varDelta h(t,x,y) - \lvert h(t,x,y) \rvert^{2} h(t,x,y), &x,y \in [-5,5], \,\, t \in [0,\pi /2]\\ h(0,x,y) &= \text{sech}(x)+0.5\text{sech}(y-2)+0.5\text{sech}(y+2), &x,y \in [-5,5]\\ h(t,-5,y) &= h(t,5,y), &t \in [0,\pi /2],\,\, y\in [-5,5]\\ h(t,x,-5) &= h(t,x,5), &t \in [0,\pi /2],\,\, x\in [-5,5]\\ \frac{\partial h(t,-5,y)}{\partial x} &= \frac{\partial h(t,5,y)}{\partial x}, &t \in [0,\pi /2],\,\, y\in [-5,5]\\ \frac{\partial h(t,x,-5)}{\partial y} &= \frac{\partial h(t,x,5)}{\partial y} &t \in [0,\pi /2],\,\, x\in [-5,5]. \end{align*}

**Fig. 12.**
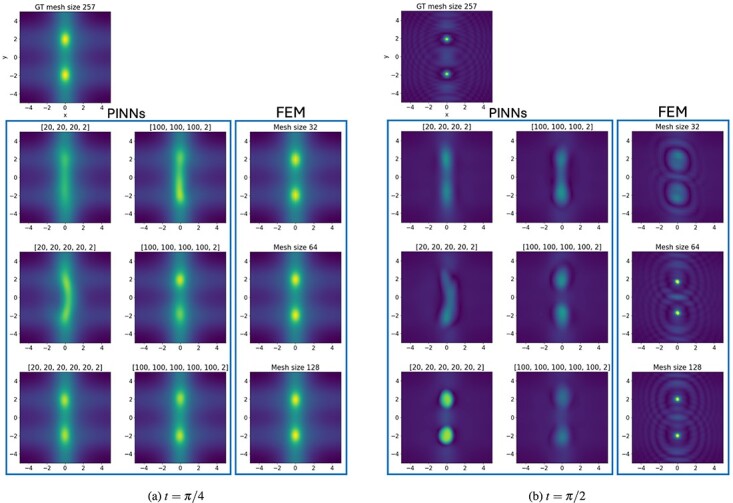
Comparison of the 2D semilinear Schrödinger solution $\lvert h(t,x) \rvert = \sqrt{u_{R}^{2}(t,x) + u_{I}^{2}(t,x)}$ at times $t = \pi /4$ and $t = \pi /2$ approximated by FEM and PINNs of different mesh and architecture sizes.

The PDE is complex-valued and we approximate the solution for $h(t,x,y) =: u_{R}(t,x,y) + \mathrm{i}u_{I}(t,x,y)$. The approximate solutions for the modulus for $\lvert h(t,x,y) \rvert = \sqrt{u_{R}^{2}(t,x,y) + u_{I}^{2}(t,x,y)}$ are shown in Fig. 12.

#### 6.2.1 FEM

Similar to the one-dimensional case of the Schrödinger equation, we split the weak formulation of the PDE (again for Dirichlet $u_{\partial } = 0$ boundaries) into its real and imaginary parts: 


\begin{align*} \int_{-5}^{5}\int_{-5}^{5} (u^{I}_{k+1}(x,y) &- u^{I}_{k}(x,y))v^{R}(x,y) \mathrm{d}x \mathrm{d}y- 0.5 \int_{-5}^{5}\int_{-5}^{5} \langle \nabla u^{R}_{k+1}(x,y), \nabla v^{R}(x,y)\rangle \mathrm{d}x \mathrm{d}y \\ &- \lvert h_{k}(x,y) \rvert^{2} \int_{-5}^{5}\int_{-5}^{5} u^{R}_{k+1}(x,y)v^{R}(x,y) \mathrm{d}x \mathrm{d}y = 0,\\ \int_{-5}^{5}\int_{-5}^{5} (u^{R}_{k+1}(x,y) &- u^{R}_{k}(x,y))v^{I}(x,y) \mathrm{d}x \mathrm{d}y + 0.5 \int_{-5}^{5}\int_{-5}^{5} \langle \nabla u^{I}_{k+1}(x,y), \nabla v^{I}(x,y)\rangle \mathrm{d}x \mathrm{d}y \\ &+ \lvert h_{k}(x,y) \rvert^{2} \int_{-5}^{5}\int_{-5}^{5} u^{I}_{k+1}(x,y)v^{I}(x) \mathrm{d}x \mathrm{d}y = 0, \end{align*}

for all test functions $v^{R},v^{I} \in H_{0}^{\varOmega }$. Time is discretized with $dt = 10^{-3}$ and the finite elements are defined on a rectangular mesh in the domain $[-5,5] \times [-5,5]$. We consider meshes with $\{(16,16),(32,32),(40,40),(64,64),(128,128)\}$ squares, each again being split into two triangles on which we define piecewise-linear finite element basis functions ($P_{1}$). We again use gmres to solve the linear system.

#### 6.2.2 PINNs

The loss functional for the neural network approximations are defined for the real and imaginary parts: 


\begin{align*} \text{Loss}(\theta) = \, &\frac{1}{N_{f}} \sum_{i = 1}^{N_{f}} (\lVert \frac{\partial u^{I}_{\theta}(t^{i}_{f},x^{i}_{f},y^{i}_{f})}{\partial t} - \epsilon \varDelta u^{R}_{\theta} (t^{i}_{f},x^{i}_{f},y^{i}_{f}) - \lvert h_{\theta}(t^{i}_{f},x^{i}_{f},y^{i}_{f}) \rvert^{2} u^{R}_{\theta}(t^{i}_{f},x^{i}_{f},y^{i}_{f}) \rVert^{2}_{2} \\ &\qquad + \lVert \frac{\partial u^{R}_{\theta}(t^{i}_{f},x^{i}_{f},y^{i}_{f})}{\partial t} + \epsilon \varDelta u^{I}_{\theta} (t^{i}_{f},x^{i}_{f},y^{i}_{f}) + \lvert h_{\theta}(t^{i}_{f},x^{i}_{f},y^{i}_{f}) \rvert^{2} u^{I}_{\theta}(t^{i}_{f},x^{i}_{f},y^{i}_{f}) \rVert^{2}_{2} )\\ &+ \frac{1}{N_{g}} \sum_{k = 1}^{N_{g}}(\lVert u^{R}_{\theta}(t^{j}_{g},-5,y^{j}_{g}) - u^{R}_{\theta}(t^{j}_{g},5,y^{j}_{g}) \rVert^{2}_{2} + \lVert u^{I}_{\theta}(t^{j}_{g},-5,y^{j}_{g}) - u^{I}_{\theta}(t^{j}_{g},5,y^{j}_{g}) \rVert^{2}_{2}\\ &\qquad + \lVert u^{R}_{\theta}(t^{j}_{g},x^{j}_{g},-5) - u^{R}_{\theta}(t^{j}_{g},x^{j}_{g},5) \rVert^{2}_{2} + \lVert u^{I}_{\theta}(t^{j}_{g},x^{j}_{g},-5) - u^{I}_{\theta}(t^{j}_{g},x^{j}_{g},5) \rVert^{2}_{2} )\\ &+ \frac{1}{N_{g}} \sum_{k = 1}^{N_{g}}\left(\left\lVert \frac{\partial u^{R}_{\theta}(t^{j}_{g},-5,y^{j}_{g})}{\partial x} - \frac{\partial u^{R}_{\theta}(t^{j}_{g},5,y^{j}_{g})}{\partial x} \right\rVert^{2}_{2} + \left\lVert \frac{\partial u^{I}_{\theta}(t^{j}_{g},-5,y^{j}_{g})}{\partial x} - \frac{\partial u^{I}_{\theta}(t^{j}_{g},5,y^{j}_{g})}{\partial x} \right\rVert^{2}_{2} \right)\\ &+ \frac{1}{N_{g}} \sum_{k = 1}^{N_{g}}\left(\left\lVert \frac{\partial u^{R}_{\theta}(t^{j}_{g},x^{j}_{g},-5)}{\partial y} - \frac{\partial u^{R}_{\theta}(t^{j}_{g},x^{j}_{g},5)}{\partial y} \right\rVert^{2}_{2} + \left\lVert \frac{\partial u^{I}_{\theta}(t^{j}_{g},x^{j}_{g},-5)}{\partial y} - \frac{\partial u^{I}_{\theta}(t^{j}_{g},x^{j}_{g},5)}{\partial y} \right\rVert^{2}_{2} \right)\\ &+ \frac{1}{N_{h}} \sum_{k = 1}^{N_{h}}\left(\lVert u^{R}_{\theta}(0,x^{k}_{h},y^{k}_{h}) - \text{sech}(x^{k}_{h}) - 0.5\text{sech}(y^{k}_{h}-2) - 0.5\text{sech}(y^{k}_{h}+2) \rVert^{2}_{2}\right), \end{align*}

for $h_{\theta }(t,x,y) = u^{R}_{\theta }(t,x,y) + \mathrm{i}u^{I}_{\theta }(t,x,y)$ the neural network with an output size 2 for the real and imaginary parts and $\theta $ the network weights that are determined by training. The loss functional is optimized based on latin hybercube sampled collocation points $(t_{f}^{i},x_{f}^{i},y_{f}^{i}) \in [0, \pi /2] \times [-5,5] \times [-5,5]$ with $N_{f} = 5,000$ points for the domain, $N_{g} = 100$ points for the boundary and $N_{h} = 100$ points for the initial condition. We chose feed-forward dense neural network in the architecture design with $\tanh $ the activation function. The results are compared for six different neural network sizes: [20,20,20,2], [100,100,100,2], [20,20,20,20,2], [100,100,100,100,2], [20,20,20,20,20,2] and [100,100,100,100,100,2]. The Adam optimizer is run for $50,000$ epochs with a learning rate of $10^{-3}$ before employing the L-BFGS optimizer.

#### 6.2.3 Results

**Fig. 13.**
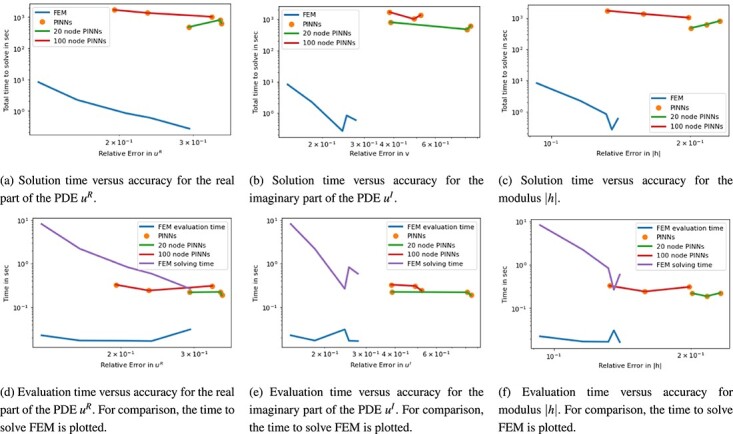
Plot for 2D Schrödinger equation of time in sec versus $\ell ^{2}$ relative error. Plots are split for the real and imaginary parts of the PDE as well as $\lvert h \rvert = \sqrt{u^{2}_{R} + u_{I}^{2}}$.

The ground truth solution was derived with FEM on a mesh of 257 cells using $P_{2}$ basis functions. The time stepping size up to $T = \pi /2$ was chosen as $dt = 5\times 10^{-4}$. A first look at the visualization of the results at an example times $t = \pi /4$ and $\pi /2$ in Fig. 12 shows that PINNs is having difficulties to recover particularly the wave-type shapes and the correct diameter of the peaks at $t = \pi /2$. Smaller network architectures are not always able to accurately separate the two peaks in the image even at earlier time instances. While PINNs are able to reproduce the main features of the PDE solution when the architecture is large enough, the examples shown lack the detailed features containing edges and discontinuities. For FEM, we observe that coarse meshes similarly fail at recovering the fine details. We also observe that the symmetry of the peaks is not preserved with a coarser mesh. However, with finer meshes, the FEM approximation appears to be good. Considering the quantified results on time versus accuracy in Fig. 13, the difference in accuracy between PINNs and FEM becomes less prudent in the real part. However, for both solving time in Figs 11(a)–13(c) and evaluation time in Figs 11(d)–13(f) FEM clearly outperforms PINNs by two to three and one order of magnitude, respectively. Taking the FEM solution time into account, we can see that PINNs slightly outperforms in their evaluation time with similar relative error. Let us also note that the PINN approximation of the imaginary part of the complex-valued PDE solution is significantly less accurate compared with the FE method.

## 7. Discussion and conclusions

After having investigated each of the PDEs on its own, let us now discuss and draw conclusions from the results as a whole. Considering the solution time and accuracy, PINNs are not able to beat FEM in our study. In all the examples that we have studied, the FEM solutions were faster at the same or at a higher accuracy.

We will now try to explain this outcome by briefly discussing the computational complexity of the FEM and of the training in PINNs. When using a multigrid method, an elliptic equation can often be solved in $O(N_{\mathrm{DoF}})$ floating point operations, where $N_{\mathrm{DoF}}$ is the number of degrees of freedom in the discretization, see, e.g. chapter 13 in Iserles (2008). We usually expect $N_{\mathrm{DoF}}$ to increase exponentially with the dimension $d$. We have neither used geometric nor algebraic multigrid methods in this work; the algorithms we used are actually slower than multigrid, but we still argue with the theoretical cost of multigrid methods. If we ignore the GPU architecture, we can estimate the complexity of a single gradient evaluation with respect to weights and biases and give one data point in the PINN as 


(7.1) \begin{align*}& O(dN_{e} + N_{l}N_{e}^{2} + N_{e}),\end{align*}

where $N_{l}\in \mathbb{N}$ describes the number of hidden layers, i.e. all layers but the input and the output layer. To simplify the presentation, we make the assumption that $N_{e}(l) = N_{e}$ constant for all layers $l = 1,..., N_{l}$ and $N_{e} \in \mathbb{N}$. The first layer is of size $d \times N_{e},$ the last one is of size $N_{e} \times 1$ and all intermediate hidden layers are of size $N_{e} \times N_{e}$. We have derived this complexity following the neat summary of the backpropagation algorithm in Higham & Higham (2019), where we note that gradient evaluations in PINNs actually require multiple backpropagations for each step, but this finite number does not increase the asymptotic complexity. When employing the Adam optimizer, the complexity cost of a single step without mini-batching is exactly the given cost in (7.1); the cost for one Adam epoch is given by $N_{f} +N_{g}+N_{h}$ times the cost in (7.1). In L-BFGS, we similarly incur the cost of one Adam epoch in each step and additionally linear cost in the size of the parameter space that does not change the asymptotics. Thus, denoting the number of Adam epochs and L-BFGS steps by $N_{\text{Adam}}$ and $N_{\text{L-BFGS}}$, respectively, the complete cost can be stated as 


\begin{align*} &O((dN_e + N_lN_e^2 + N_e)(N_{\text{Adam}} + N_{\text{L-BFGS}})(N_f + N_g + N_h)).\end{align*}

This appears to scale nicely in dimension $d$, but naturally $N_{l}, N_{e}, N_{\text{Adam}}, N_{\text{L-BFGS}}, N_{f}, N_{g}, N_{h}$ may depend on $d$. The usual GPU acceleration of backpropagation reduces the computational cost of these operations considerably. Moreover, especially in high dimensions, we would usually anticipate to be able to choose number and size of layers such that the combined size of weight matrices $dN_{e} + N_{l}N_{e}^{2} + N_{e}$ and bias vectors $N_{l}N_{e} +1$ is considerably smaller than the degrees of freedom $N_{\mathrm{DoF}}$ in a usual FEM approximation. The largest contribution to the computational cost is likely the large number of necessary Adam and L-BFGS steps that are necessary in the non-convex learning problem.

After having solved the PDE, PINNs are sometimes faster at the pointwise evaluation of the respective solution: we were only able to show this in the 3D Poisson test, where the FEM evaluation after solution was rather slow. So when needing to evaluate a PDE on a very fine grid, one could consider solving a PINN. Although, in our examples, the solution time of FEM was so much faster that continued solving the PDE with FEM on different adapted grids would likely still be considerably faster than solving and evaluating the PINN. In addition, we believe that the evaluation time in FEM could be significantly sped up using more appropriate interpolation methodology, such as Lin & Lee (2005); Zhang *et al.* (2021).

We were particularly surprised that PINNs had difficulties with the Allen–Cahn equation using a small $\varepsilon $. This might be due to the close-to-discontinuous behaviour at the diffuse interface, which may be more pronounced in the strong form of the PDE that PINNs aim to solve. We anticipated that PINNs would outperform FEM: the solution of an Allen–Cahn equation has very much the flavour of a classification (see Budd *et al.* (2021)) at which neural networks excel; FEM requires a very fine grid to resolve the diffuse interface. A similar case in which we were surprised that PINNs did not outperform FEM was the Schrödinger 2D examples, where the PDE solution, again, contains very finely structured areas. In both these cases an adaptive PINNs approach or variational PINNs (Kharazmi *et al.*, 2019) might help. The latter would then also allow activation functions that have weak derivatives.

An aspect of the evaluation time that we have not considered throughout this work is the possibility of solving parameterized PDEs with neural networks, the so-called operator approximators. See, for instance, Fourier Neural Operators (Li *et al.*, 2021) and DeepONets (Lu *et al.*, 2021a). While FEM requires continued solutions of PDEs when changing the parameters, neural networks can take parameters as additional inputs and be trained throughout all of them—we have seen that PINN evaluations are sometimes faster than FEM solutions. They have been shown to work well as surrogates if the PDE needs to be solved sufficiently often; see also Tanyu *et al.* (2023). In a future work, those should be compared with classical methods for parameterized PDEs, such as reduced bases (Quarteroni *et al.*, 2016) or low-rank tensor methods (Kressner & Tobler, 2011). Again, the time of the offline phase in which the parametric model is constructed or trained has to be considered carefully.

PINNs were good at the transition into higher dimensions: there is no increment in computational cost from the Poisson equation in 2D and 3D. The 3D Poisson equation we consider is completely isotropic, which is a structure the PINN appears to be able to use easily. A basic FEM approach cannot make use of it. In a more complicated anisotropic or even regular spatially varying setting, we would anticipate a similar computational cost and speed for FEM, but cannot make any predictions for PINNs. This hints at the efficiency of PINNs in certain high-dimensional settings, in which classical techniques (such as FEM) are prohibitively expensive. This has been considered in, e.g. Kunisch & Walter (2021) and Hu *et al.* (2024). In general, PINNs open up many interesting new research directions, especially when employed to solve such high-dimensional PDEs or when combining PDEs and data. The analysis of PINNs is both very challenging and highly interesting. Our study suggests that for certain classes of PDEs for which classical methods are applicable, PINNs are not able to outperform those.
